# A comparative study of reduced dose alemtuzumab in matched unrelated donor and related donor reduced intensity transplants

**DOI:** 10.1111/bjh.13239

**Published:** 2015-01-29

**Authors:** Laura Jardine, Amy Publicover, Venetia Bigley, Geoff Hale, Kim Pearce, Anne Dickinson, Graham Jackson, Matthew Collin

**Affiliations:** ^1^ Institute of Cellular MedicineNewcastle UniversityNewcastle upon TyneUK; ^2^ Newcastle upon Tyne Hospitals NHS Foundation TrustNewcastle upon TyneUK; ^3^ University Hospital SouthamptonSouthamptonUK; ^4^ Mablyte LtdReadingUK

**Keywords:** bone marrow transplantation, T cell depletion, graft‐versus‐host disease

## Abstract

*In vivo* T cell depletion with 100 mg alemtuzumab prevents graft‐versus‐host disease (GVHD) in reduced intensity conditioned transplants but is associated with delayed immune reconstitution, a higher risk of infection and relapse. De‐escalation studies have shown that a reduced dose of 30 mg is as effective as 100 mg in preventing GVHD in matched related donor (MRD) transplants. Dose reduction in matched unrelated donor (MUD) transplants is feasible but the comparative efficacy of alemtuzumab in this setting is not known and opinions vary widely concerning the optimal level of GVHD prophylaxis that should be achieved. Through retrospective analysis we made an objective comparison of MUD transplants receiving an empirically reduced dose of 60 mg, with MRD transplants receiving a 30 mg dose. We observed proportionate levels of alemtuzumab according to dose but an inverse relationship with body surface area particularly in MRD transplants. MUD transplants experienced more acute and chronic GVHD, higher T cell chimerism, more sustained use of ciclosporin and less need for donor lymphocyte infusion than MRD transplants. Thus, doubling the dose of alemtuzumab to 60 mg did not provide equivalent prevention of GVHD after MUD transplant although there was no difference in non‐relapse mortality or survival compared with MRD transplants.

Alemtuzumab (CAMPATH 1H), a humanised immunoglobulin G1 (IgG1) monoclonal antibody against CD52, is a well‐established agent for effecting *in vivo* T cell depletion in the setting of allogeneic haematopoietic stem cell transplantation. By reducing the number of both donor and recipient T cells, it allows engraftment, while reducing the incidence of acute and chronic graft‐versus‐host disease (GVHD) (Kottaridis *et al*, 2000; Chakraverty *et al*, 2002). Conventional alemtuzumab dosing (100 mg in five daily doses of 20 mg) has been shown to ablate acute and chronic GVHD almost completely in fludarabine and melphalan (FM) conditioned matched related donor (MRD) and matched unrelated donor (MUD) transplants (Kottaridis *et al*, 2000; Chakraverty *et al*, 2002). Subsequent studies have reported lower rates of chronic GVHD with equivalent overall survival compared with GVHD prophylaxis with methotrexate or mycophenolate mofetil (Perez‐Simon *et al*, 2002; Delgado *et al*, 2008; van Besien *et al*, 2009; Malladi *et al*, 2009). This is achieved, however, at the expense of delayed immune reconstitution (Morris *et al*, 2003; Dodero *et al*, 2005), an increase in opportunistic infections (Chakrabarti *et al*, 2002a,b; Perez‐Simon *et al*, 2002; Avivi *et al*, 2004) and higher incidence of mixed chimerism, which may impair graft‐versus‐leukaemia responses (Mackinnon *et al*, 1994).

The risks of T cell depletion can be ameliorated by donor lymphocyte infusions (DLIs) (Peggs *et al*, 2004, 2007; Bloor *et al*, 2008; Thomson *et al*, 2010; Mohamedbhai *et al*, 2012; Liga *et al*, 2013) but there is a need to define minimum levels of T cell depletion that provide effective protection from GVHD in different settings. A formal dose de‐escalation study in MRD transplants concluded that a single alemtuzumab dose of 30 mg on day −1 is sufficient to prevent GVHD (Chakraverty *et al*, 2010) but equivalent studies in MUD transplants are lacking. Favourable outcomes have been reported in mixed cohorts of MRD and MUD transplants for a wide range of alemtuzumab doses from 50 mg to as little as 10 mg (Faulkner *et al*, 2004; Khouri *et al*, 2004; Tholouli *et al*, 2008; Bertz *et al*, 2009; Spyridonidis *et al*, 2011; Gartner *et al*, 2013; Potter *et al*, 2014). These are difficult to interpret: first, they involve mixed cohorts of MRD and MUD transplants; and second, the definition of ‘adequate’ control of GHVD is entirely subjective with some centres tolerating much higher rates of acute GVHD and extensive chronic GVHD than others.

In the absence of a formal de‐escalation study in MUD transplants and lack of rational criteria to define adequate GVHD prophylaxis, we compared an empirically reduced dose of 60 mg alemtuzumab in MUD transplants, with 30 mg for MRD transplants, previously defined by formal study (Chakraverty *et al*, 2010). This offers an objective assessment of the potential of alemtuzumab to modify unrelated donor alloreactivity, relative to the level of a sibling donor.

## Methods

### Study design and ethics

The study included sequential patients transplanted with FM conditioning, following the introduction of de‐escalated alemtuzumab dosing for MRD and MUD transplants in 2006. Clinical records were interrogated for GVHD, survival, chimerism testing and ciclosporin levels. All patients gave consent for clinical follow‐up and post‐transplant serum sampling for research purposes, according to protocols approved by the local research ethics committee of Northumberland and North Tyneside.

### Conditioning regimen and alemtuzumab dosing

All patients received fludarabine 30 mg/m^2^ per day from day −7 to −3 and melphalan 140 mg/m^2^ day −2. Alemtuzumab 30 mg was given on day −2 for MRD transplants and on days −4 and −2 for MRD transplants (total 60 mg). Day −2 was preferred rather than day −1 because kinetic studies indicated a steep decline in levels in the first 24 h after infusion (Morris *et al*, 2003). We reasoned that the timing of stem cell infusion, which is not well controlled, would have less effect on the level of T cell depletion if we allowed 48 h following alemtuzumab adminstration, at which point alemtuzumab levels would be more stable. A minimum CD34^+^ stem cell dose of 4 × 10^6^/kg was infused in all patients. Historical patients treated with 100 mg of alemtuzumab received 20 mg daily from day −7 to −3, inclusive.

### Post‐transplant monitoring and management

Graft‐versus‐host disease prophylaxis was ciclosporin given at 2·5 mg/kg twice daily from day −1 (with a target of 200–300 μg/l). Ciclosporin levels were monitored twice weekly by enzyme‐linked immunosorbent assay. Tapering began at day +30 in the absence of GVHD. All patients received chemoprophylaxis against *Pneumocystis jirovecii* and Varicella Zoster. Serum cytomegalovirus (CMV) copy number was assessed twice weekly by polymerase chain reaction (PCR) and pre‐emptive therapy was initiated if copy number rose above >10^4^. Peripheral blood and bone marrow chimerism was tested by short tandem repeat PCR on unfractionated bone marrow and magnetically‐selected CD3^+^ and CD15^+^ fractions of peripheral blood. Monitoring took place monthly until day +100 and 3‐monthly thereafter. A schedule of DLIs, escalating by half‐log increments every 3 months was instituted for persistent partial chimerism, beginning at 3 × 10^5^/kg at 6 months. Patients with early relapse received 10‐fold higher doses at 4–6 weekly intervals. Clinical assessment of acute GVHD was performed at least once weekly in the first 100 d using modified Glucksberg criteria (Hunt *et al*, 2001). Incidence of chronic GVHD was assessed at 1 year post‐transplant and graded using both Seattle and National Institutes of Health criteria (Shulman *et al*, 1980; Filipovich *et al*, 2005). Body surface area was estimated by the method of Dubois (Dubois & Dubois, 1916).

### Serum alemtuzumab measurement

Post‐transplant serum samples were available from 20 MRD recipients and 13 MUD recipients at day +1 and 11 MRD recipients and nine MUD recipients at day 3–4. Alemtuzumab concentration was measured as previously described by a validated flow cytometry assay (Rebello & Hale, 2002).

### Statistical methods

Intergroup analyses were performed with Student's *t* test for continuous variables, Mann–Whitney test for proportions and Chi‐square for contingency. The three group‐comparison of serum alemtuzumab levels used one‐way analysis of variance (anova). Multiple *t*‐tests used for intergroup analysis of serum ciclosporin levels over time were subjected to sequential Bonferroni correction. Overall survival was analysed by the Kaplan Meier method with log–rank test to compare survival curves. Relapse and non‐relapse mortality were analysed by the cumulative incidence method with relapse as a competing event for non‐relapse mortality (NRM) and vice‐versa. Cumulative incidence curves were compared with Gray's test. Hazard ratios were calculated by Cox Regression modelling. Graphing and statistical analyses were performed with graphpad prism version 6.0 (GraphPad Software, Inc. La Jolla, CA, USA) except for cumulative incidence, which was performed with r version 3.1.0 and Cox Regression, which was performed with spss version 21 (IBM United Kingdom Ltd., Portsmouth, UK). *P* < 0·05 was recorded as significant.

## Results

### Patient characteristics

**Figure 1:**
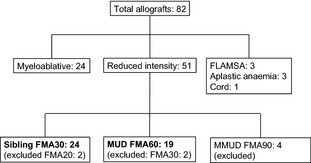
Consort diagram summarising patients included in the study. FMA: fludarabine, melphalan, alemtuzumab (dose in mg); FLAMSA: fludarabine, cytarabine, amsacrine (with cyclophosphamide and total body irradiation transplant); MUD, matched unrelated donor; MMUD, mismatched unrelated donor (<9/10 antigen‐mismatched MUD).

From May 2006 to May 2009, 24 patients received MRD transplants and 19 received MUD transplants. Over this period a total of 51 patients were transplanted with reduced intensity conditioning (Fig 1). Eight were not included in the study because they were transplanted with different doses of alemtuzumab (two MRD and two MUD transplants) or the MUD transplants were less than 9/10 human leucocyte antigen (HLA)‐matched (four patients). Patients included in the study were well matched for age, transplant indication and donor/recipient CMV status (Table 1). All MRD recipients were 12/12 HLA‐matched. Seventeen of the unrelated donors were 10/10 matched at high resolution. Two donors were 9/10 matched due to single allele mismatches in HLA‐Cw.

**Table 1:** Patient characteristics

Patient baseline data	FMA 30 *n* = 24	FMA 60 *n* = 19	*P*
Age at transplant (years), mean ± SEM	45 ± 1·3	48 ± 2·9	ns
Gender direction of transplant *n* (%)			
Male to male	8 (33)	10 (53)	ns
Male to female	6 (25)	6 (32)	
Female to male	7 (29)	1 (5)	
Female to female	3 (13)	2 (11)	
Transplant indication			
AML	14 (58)	9 (47)	ns
MDS	2 (8)	1 (5)	
NHL	6 (25)	2 (11)	
HL	1 (4)	3 (16)	
CML	1 (4)	0	
Myeloma	0	3 (16)	
Other	0	1 (5)	
Stem cell source			
PBSC	18 (75)	15 (79)	ns
BM	6 (25)	4 (21)	
CMV (donor/recipient)			
Negative/positive	10 (42)	5 (26)	ns
Positive/positive	5 (21)	6 (32)	
Positive/negative	2 (8)	2 (11)	
Negative/negative	5 (21)	6 (32)	

FMA, fludarabine, melaphalan, alemtuzumab; SEM, standard error of the mean; AML, acute myeloid leukaemia; MDS, myelodysplastic syndrome; HNL, non‐Hodgkin lymphoma; HL, Hodgkin lymphoma; CML, chronic myeloid leukaemia; PBSC. Peripheral blood stem cells; BM, bone marrow; CMV, cytomegalovirus.

### Serum Alemtuzumab levels

**Figure 2:**
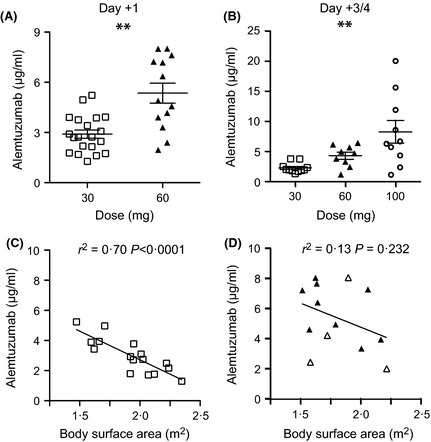
(A) Comparison of mean serum alemtuzumab level on day +1 in matched related donor (MRD) recipients of 30 mg and matched unrelated donor (MUD) recipients of 60 mg **P = 0·0002. (B) Comparison of mean serum alemtuzumab level on day +3–4 in MRD recipients of 30 mg, MUD recipients of 60 mg and historical controls receiving 100 mg alemtuzumab **P = 0·0028. (C) Correlation between body surface area and serum alemtuzumab in MRD recipients of 30 mg; open symbols indicate bone marrow (BM); closed symbols indicate peripheral blood stem cell (PBSC) grafts. (D) Correlation between body surface area (Dubois) and serum alemtuzumab in MUD recipients of 60 mg. Open symbols indicate BM; closed symbols indicate PBSC grafts.

Serum had been collected on days +1, +3–4 and +7 for immunomonitoring and a selection of samples was available for measurement of alemtuzumab levels. On day +1, the mean ± standard deviation (SD) serum alemtuzumab level was 2·9 ± 1·1 μg/ml in MRD transplants (30 mg) compared with 5·4 ± 2·2 μg/ml in MUD transplants (60 mg); (*P* = 0·0002; Fig 2A). A collection of samples taken on day +3–4 was also available to compare dose‐reduced transplants with historical patients receiving 100 mg alemtuzumab (Fig 2B). The serum alemtuzumab levels for both MRD and MUD transplants were significantly lower with mean ± SD of 2·3 ± 0·8 and 4·3 ± 1·8 μg/ml, respectively, compared with 8·2 ± 6·0 μg/ml for recipients of 100 mg of alemtuzumab (*P* < 0·0028). Alemtuzumab dosing regimens in adults do not usually take into account the size of the patient but an inverse relationship was observed between alemtuzumab level and body surface area (BSA) in both cohorts, reaching significance in MRD transplants (*r* ^2^ = 0·70; *P* < 0·0001; Fig 2C,D).

### Chimerism

**Figure 3:**
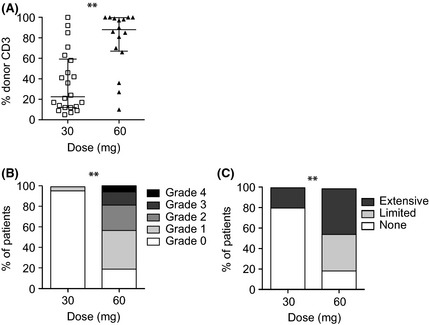
(A) Comparison of median peripheral blood T cell chimerism at day 100 in matched unrelated donor (MRD) recipients of 30 mg and matched unrelated donor (MUD) recipients of 60 mg alemtuzumab **P = 0·0002. (B) Incidence and severity of acute graft‐versus‐host disease (GVHD) in MRD recipients of 30 mg and MUD recipients of 60 mg alemtuzumab; **P < 0·0001. (C) Incidence and severity of chronic GVHD in MRD recipients of 30 mg and MUD recipients of 60 mg alemtuzumab; **P < 0·0001.

Chimerism data were available for all (*n* = 39) patients evaluable at day +100. Median myeloid engraftment (CD15^+^ cells) was 100% in both cohorts (not shown). Donor T cell engraftment (CD3^+^ cells) was significantly higher in MUD than MRD recipients (median ± interquartile range: 88 ± 33 compared with 21 ± 46; *P* = 0·0002; Fig 3A). As a result of treating partial T cell chimerism pre‐emptively in patients surviving more than 6 months, fewer MUD patients received DLI (4/19; seven doses in total) compared with MRD patients (20/24; 31 doses in total), *P* < 0·0001.

### Graft‐versus‐host disease

Overall, 13 out of 16 (81%) MUD recipients surviving to day +100 experienced acute GVHD compared with 1 out of 23 (4%) MRD recipients, *P* < 0·0001. GVHD grade II or greater occurred only in MUD recipients (44% vs. 0%, *P* < 0·0001) (Fig 3B). Similarly, chronic GVHD was more common in MUD recipients (55% vs. 20%; *P* < 0·0001) and more often extensive (Fig 3C).

### Immunosuppression

**Figure 4:**
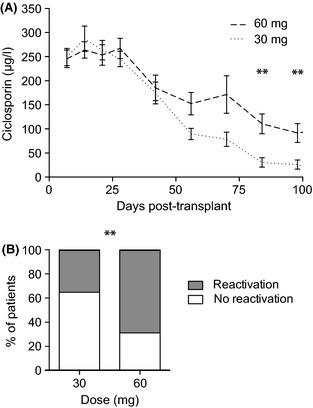
(A) Comparison of mean (± SEM) ciclosporin levels in matched related donor (MRD) patients receiving 30 mg (dotted line) versus matched unrelated donor (MUD) patients receiving 60 mg alemtuzumab. Significant differences between means after Bonferroni correction; **P = 0·0007 at day +84 and *P* = 0·0028 at day +98. (B) Incidence of cytomegalovirus reactivation in seropositive recipients of 30 mg (MRD) and 60 mg (MUD) alemtuzumab, **P < 0·0001.

Ciclosporin was tapered in the absence of GVHD from day +30. Increased GVHD in the MUD recipients was reflected by diverging mean serum ciclosporin concentrations from day +50 (Fig 4A). Overall, 13/16 (81%) MUD recipients still received ciclosporin at day +100 compared with 5/23 (22%) MRD recipients. Ciclosporin was completely withdrawn from all MRD recipients by day 128 but 7/16 (44%) MUD recipients still required immunosuppression.

### Cytomegalovirus reactivation

Cytomegalovirus reactivation was seen in 15 (35%) patients overall. The reactivation rates in CMV seropositive individuals were 69% in MUD recipients and 35% in MRD recipients, *P* < 0·0001 (Fig 4B).

### Relapse mortality, non‐relapse mortality and overall survival

**Figure 5:**
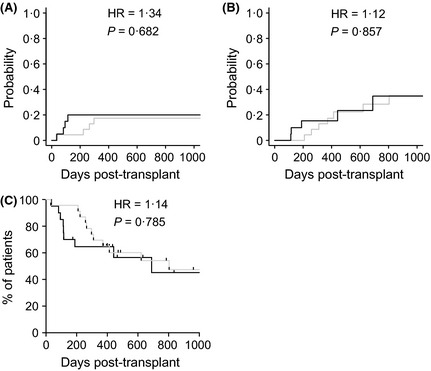
(A) Cumulative incidence of non‐relapse mortality. (B) Cumulative incidence of relapse. (C) Kaplan Meier curves of overall survival. Grey lines show matched related donor (MRD) recipients of 30 mg alemtuzumab; black lines show matched unrelated donor (MUD) recipients of 60 mg alemtuzumab. HR, Hazard ratio.

Estimates for cumulative incidence of non‐relapse mortality at day +100 and 2 years were 4% (95% confidence interval [CI] 2–19%) and 17% (5–35%) for MRD recipients and 15% (4–34%) and 20% (6–40%) for MUD recipients (Fig 5A). The hazard ratio for NRM in MUD versus MRD recipients was 1·337 (95% CI 0·333–5·370), *P* = 0·682. Cumulative incidence of relapse at 2 years was 28% (11–50%) in MRD recipients and 35% (10–62%) in MUD recipients (Fig 5B). The hazard ratio for relapse in MUD versus MRD recipients was 1·116 (95% CI 0·353–3·527), *P* = 0·857. By Kaplan–Meier analysis, the median survival was 804 d in MRD and 609 in MUD transplant recipients. Overall survival proportions at 2 years were 54% (31–72%) and 45% (18–69%) in MRD and MUD recipients, respectively (Fig 5C). The hazard ratio for death in MUD versus MRD recipients was 1·135 (95% CI 0·455–2·831), *P* = 0·785.

## Discussion

The optimal dose of alemtuzumab for *in vivo* T cell depletion of reduced intensity conditioned transplantation is controversial and depends upon subjective judgments about the level of GVHD that tolerable or desirable, the perceived risk of relapse and the willingness of physicians to use pre‐emptive DLI to bolster post‐transplant immune reconstitution. The overall survival has been reported to be comparable between patients receiving widely ranging levels of *in vivo* T cell depletion (Perez‐Simon *et al*, 2002; Delgado *et al*, 2008; van Besien *et al*, 2009; Malladi *et al*, 2009). The use of alemtuzumab is generally associated with higher relapse risk but less non‐relapse death and proponents argue that the quality of survivorship is improved by the prevention of extensive chronic GVHD (Perez‐Simon *et al*, 2002), particularly in the T cell compartment.

Here we have evaluated 60 mg dosing for MUD transplants relative to the 30 mg dose, defined as optimal for stringent control of GVHD in MRD transplants (Chakraverty *et al*, 2010). Monitoring of serum indicates that much lower levels are achieved than with 100 mg of alemtuzumab and that there is a predictable difference between 30 mg for MRD and 60 mg for MUD transplants, at day +1. We elected to give alemtuzumab 1 d earlier than Chakraverty *et al* (2010), at day −2, and the dose measured at day +1 was 30–40% lower than that reported on day 0 by these authors (2·9 μg/ml for 30 mg and 5·4 μg/ml for 60 mg in this study compared with approximately 5 and 7·5 μg/ml, respectively). However, this did not appear to increase the incidence of acute GVHD grades II–IV in our MRD cohort (both studies <5%). We also demonstrated a significant inverse relationship with BSA for MRD transplants and a trend in the same direction for MUD transplants. Metre‐squared dosing is common in paediatric practise (Dodero *et al*, 2005; Shah *et al*, 2007) and it is possible that more predictable outcomes would be observed if a similar approach was taken in adults (Morris *et al*, 2003; Chakraverty *et al*, 2010). The use of one 30 mg dose (two 30 mg doses for MUD transplants) in our schedule also makes economic sense, as 30 mg is the presentation vial size of the drug. One possible drawback is that the infusional toxicity of alemtuzumab may be higher with a single dose than with several fractionated doses, although we did not observe major difficulties with this. Prior commencement of fludarabine (day −7) before the first dose of alemtuzumab (day −4) may have reduced the incidence of infusional toxicity in these patients.

Our study is limited by the retrospective nature and relatively small number of patients transplanted over the period of observation. A prospective study would have been preferred but reduced alemtuzumab dosing in MUD transplants was already common practise, making a large prospective study, similar to that performed in MRD transplants, difficult to conduct. Comparison with the sibling cohort receiving 30 mg of alemtuzumab, rather than the historical MUD cohort receiving 100 mg, was motivated by the fact that sibling 30 mg and MUD 60 mg could be compared contemporaneously and that the most carefully annotated dose regime in the literature is the 30 mg dose given to siblings. The power of the study to detect differences in survival is rather low as indicated by wide confidence intervals in the hazard ratios. However, our primary aim was to report outcomes related to the control of GVHD. The observation that overall survival remains relatively constant with different levels of GVHD prophylaxis has been reported in several other studies (Perez‐Simon *et al*, 2002; Delgado *et al*, 2008; van Besien *et al*, 2009; Malladi *et al*, 2009).

Mixed cohorts of MRD and MUD transplant patients treated with lower doses of alemtuzumab have satisfactory outcomes overall (Faulkner *et al*, 2004; Khouri *et al*, 2004; Tholouli *et al*, 2008; Bertz *et al*, 2009; Spyridonidis *et al*, 2011; Gartner *et al*, 2013; Potter *et al*, 2014). In a large series of 58 MRD and 69 MUD transplants subjected to a reducing regimen of 40, 20 and 10 mg of alemtuzumab, it was possible to infer differences between the response of MRD and MUD patients to alemtuzumab (Bertz *et al*, 2009). For example, MUD transplants receiving 40 mg experienced 27% grade II–IV GVHD compared with MRD patients receiving 10 mg who only experienced 10% grade II–IV. This suggests that unrelated donors cause significantly more alloreactivity in this range of alemtuzumab dosing. In support of this, we found that 44% of MUD transplants experienced grade II–IV acute GVHD after 60 mg of alemtuzumab compared with 0% of MRD patients after 30 mg of alemtuzumab. Both reports are consistent in finding more acute and chronic GVHD with MUD transplants, even when alemtuzumab dosing was increased by several fold.

In keeping with higher rates of GVHD, we observed higher donor T cell chimerism at day 100 in MUD transplants and recorded lower use of DLI compared with MRD transplants managed with the same pre‐emptive DLI policy. Higher indices of alloreactivity in MUD transplants were not due to more aggressive withdrawal of immunosuppression, as higher ciclosporin levels were maintained in these patients after day 50. Survival, relapse and non‐relapse outcomes were comparable as in other studies. One limitation of this report is that we did not record quality of life, which may have been relatively impaired in MUD transplants owing to a higher rate of extensive chronic GVHD.

Our data suggest that 60 mg of alemtuzumab does not achieve equivalent control of GVHD compared with 30 mg in MRD transplants. However, it may be naïve to assume that an ‘equivalent’ dose can be reached by simple titration. Review of MUD transplant patients receiving the original 100 mg dose indicates a 20% incidence of acute GVHD grades II–IV (Mead *et al*, 2010). Notably, this level of acute GVHD is still above that reported for MRD transplants with 30 mg dose (0–5%) and only marginally increases to 22% when mismatching is permitted in unrelated donors (Mead *et al*, 2010). These observations suggest that the relationship between alemtuzumab dose and antigenic disparity is non‐linear and that it may not be possible to achieve equivalent GVHD prophylaxis between MUD and MRD transplants simply by dose adjustment. Fortunately, as many studies illustrate, overall survival often remains stable despite significant variation in the burden of GVHD (Perez‐Simon *et al*, 2002; Delgado *et al*, 2008; van Besien *et al*, 2009; Malladi *et al*, 2009). Further improvements in outcome may yet be achievable by paying close attention to individualised dosing of alemtuzumab based on BSA, expression of CD52 by malignant cells and the risk of graft rejection or relapse (Khouri *et al*, 2004; Chakraverty *et al*, 2010; Mead *et al*, 2010). Attention to long‐term quality of life measurements is also paramount.

## Authorship

Laura Jardine performed the research, analysed the data and wrote the paper. Amy Publicover analysed data and wrote the paper. Venetia Bigley acquired clinical data. Geoff Hale performed research and contributed data. Anne Dickinson interpreted data. Graham Jackson acquired clinical data. Matthew Collin designed the research study, analysed data and wrote the manuscript.

## Conflict of interest

The authors have no conflicts of interest to declare.

## References

[bjh13239-bib-0001] Avivi, I. , Chakrabarti, S. , Kottaridis, P. , Kyriaku, C. , Dogan, A. , Milligan, D.W. , Linch, D. , Goldstone, A.H. & Mackinnon, S. (2004) Neurological complications following alemtuzumab‐based reduced‐intensity allogeneic transplantation. Bone Marrow Transplantation, 34, 137–142. 15235576 10.1038/sj.bmt.1704538

[bjh13239-bib-0002] Bertz, H. , Spyridonidis, A. , Wasch, R. , Grullich, C. , Egger, M. & Finke, J. (2009) A novel GVHD‐prophylaxis with low‐dose alemtuzumab in allogeneic sibling or unrelated donor hematopoetic cell transplantation: the feasibility of deescalation. Biology of Blood and Marrow Transplantation, 15, 1563–1570. 19896080 10.1016/j.bbmt.2009.08.002

[bjh13239-bib-0003] van Besien, K. , Kunavakkam, R. , Rondon, G. , de Lima, M. , Artz, A. , Oran, B. & Giralt, S. (2009) Fludarabine‐melphalan conditioning for AML and MDS: alemtuzumab reduces acute and chronic GVHD without affecting long‐term outcomes. Biology of Blood and Marrow Transplantation, 15, 610–617. 19361753 10.1016/j.bbmt.2009.01.021 PMC4348112

[bjh13239-bib-0004] Bloor, A.J. , Thomson, K. , Chowdhry, N. , Verfuerth, S. , Ings, S.J. , Chakraverty, R. , Linch, D.C. , Goldstone, A.H. , Peggs, K.S. & Mackinnon, S. (2008) High response rate to donor lymphocyte infusion after allogeneic stem cell transplantation for indolent non‐Hodgkin lymphoma. Biology of Blood and Marrow Transplantation, 14, 50–58. 18158961 10.1016/j.bbmt.2007.04.013

[bjh13239-bib-0005] Chakrabarti, S. , Mackinnon, S. , Chopra, R. , Kottaridis, P.D. , Peggs, K. , O'Gorman, P. , Chakraverty, R. , Marshall, T. , Osman, H. , Mahendra, P. , Craddock, C. , Waldmann, H. , Hale, G. , Fegan, C.D. , Yong, K. , Goldstone, A.H. , Linch, D.C. & Milligan, D.W. (2002a) High incidence of cytomegalovirus infection after nonmyeloablative stem cell transplantation: potential role of Campath‐1H in delaying immune reconstitution. Blood, 99, 4357–4363. 12036862 10.1182/blood.v99.12.4357

[bjh13239-bib-0006] Chakrabarti, S. , Avivi, I. , Mackinnon, S. , Ward, K. , Kottaridis, P.D. , Osman, H. , Waldmann, H. , Hale, G. , Fegan, C.D. , Yong, K. , Goldstone, A.H. , Linch, D.C. & Milligan, D.W. (2002b) Respiratory virus infections in transplant recipients after reduced‐intensity conditioning with Campath‐1H: high incidence but low mortality. British Journal of Haematology, 119, 1125–1132. 12472597 10.1046/j.1365-2141.2002.03992.x

[bjh13239-bib-0007] Chakraverty, R. , Peggs, K. , Chopra, R. , Milligan, D.W. , Kottaridis, P.D. , Verfuerth, S. , Geary, J. , Thuraisundaram, D. , Branson, K. , Chakrabarti, S. , Mahendra, P. , Craddock, C. , Parker, A. , Hunter, A. , Hale, G. , Waldmann, H. , Williams, C.D. , Yong, K. , Linch, D.C. , Goldstone, A.H. & Mackinnon, S. (2002) Limiting transplantation‐related mortality following unrelated donor stem cell transplantation by using a nonmyeloablative conditioning regimen. Blood, 99, 1071–1078. 11807015 10.1182/blood.v99.3.1071

[bjh13239-bib-0008] Chakraverty, R. , Orti, G. , Roughton, M. , Shen, J. , Fielding, A. , Kottaridis, P. , Milligan, D. , Collin, M. , Crawley, C. , Johnson, P. , Clark, A. , Parker, A. , Bloor, A. , Pettengell, R. , Snowden, J. , Pettitt, A. , Clark, R. , Hale, G. , Peggs, K. , Thomson, K. , Morris, E. & Mackinnon, S. (2010) Impact of in vivo alemtuzumab dose before reduced intensity conditioning and HLA‐identical sibling stem cell transplantation: pharmacokinetics, GVHD, and immune reconstitution. Blood, 116, 3080–3088. 20587785 10.1182/blood-2010-05-286856

[bjh13239-bib-0009] Delgado, J. , Pillai, S. , Benjamin, R. , Caballero, D. , Martino, R. , Nathwani, A. , Lovell, R. , Thomson, K. , Perez‐Simon, J.A. , Sureda, A. , Kottaridis, P. , Vazquez, L. , Peggs, K. , Sierra, J. , Milligan, D. & Mackinnon, S. (2008) The effect of in vivo T cell depletion with alemtuzumab on reduced‐intensity allogeneic hematopoietic cell transplantation for chronic lymphocytic leukemia. Biology of Blood and Marrow Transplantation, 14, 1288–1297. 18940684 10.1016/j.bbmt.2008.09.001

[bjh13239-bib-0010] Dodero, A. , Carrabba, M. , Milani, R. , Rizzo, E. , Raganato, A. , Montefusco, V. , Farina, L. , Milanesi, M. , Longoni, P. , Carlo‐Stella, C. & Corradini, P. (2005) Reduced‐intensity conditioning containing low‐dose alemtuzumab before allogeneic peripheral blood stem cell transplantation: graft‐versus‐host disease is decreased but T‐cell reconstitution is delayed. Experimental Hematology, 33, 920–927. 16038785 10.1016/j.exphem.2005.05.009

[bjh13239-bib-0011] Dubois, D. & Dubois, E.F. (1916) A formula to estimate the approximate surface area if height and weight be known. Archives of Internal Medicine, 17, 863–871.

[bjh13239-bib-0012] Faulkner, R.D. , Craddock, C. , Byrne, J.L. , Mahendra, P. , Haynes, A.P. , Prentice, H.G. , Potter, M. , Pagliuca, A. , Ho, A. , Devereux, S. , McQuaker, G. , Mufti, G. , Yin, J.L. & Russell, N.H. (2004) BEAM‐alemtuzumab reduced‐intensity allogeneic stem cell transplantation for lymphoproliferative diseases: GVHD, toxicity, and survival in 65 patients. Blood, 103, 428–434. 12969983 10.1182/blood-2003-05-1406

[bjh13239-bib-0013] Filipovich, A.H. , Weisdorf, D. , Pavletic, S. , Socie, G. , Wingard, J.R. , Lee, S.J. , Martin, P. , Chien, J. , Przepiorka, D. , Couriel, D. , Cowen, E.W. , Dinndorf, P. , Farrell, A. , Hartzman, R. , Henslee‐Downey, J. , Jacobsohn, D. , McDonald, G. , Mittleman, B. , Rizzo, J.D. , Robinson, M. , Schubert, M. , Schultz, K. , Shulman, H. , Turner, M. , Vogelsang, G. & Flowers, M.E. (2005) National Institutes of Health consensus development project on criteria for clinical trials in chronic graft‐versus‐host disease: I. Diagnosis and staging working group report. Biology of Blood and Marrow Transplantation, 11, 945–956. 16338616 10.1016/j.bbmt.2005.09.004

[bjh13239-bib-0014] Gartner, F. , Hieke, S. , Finke, J. & Bertz, H. (2013) Lowering the alemtuzumab dose in reduced intensity conditioning allogeneic hematopoietic cell transplantation is associated with a favorable early intense natural killer cell recovery. Cytotherapy, 15, 1237–1244. 23993297 10.1016/j.jcyt.2013.05.016

[bjh13239-bib-0015] Hunt, S.C. , Abkevich, V. , Hensel, C.H. , Gutin, A. , Neff, C.D. , Russell, D.L. , Tran, T. , Hong, X. , Jammulapati, S. , Riley, R. , Weaver‐Feldhaus, J. , Macalma, T. , Richards, M.M. , Gress, R. , Francis, M. , Thomas, A. , Frech, G.C. , Adams, T.D. , Shattuck, D. & Stone, S. (2001) Linkage of body mass index to chromosome 20 in Utah pedigrees. Human Genetics, 109, 279–285. 11702208 10.1007/s004390100581

[bjh13239-bib-0016] Khouri, I.F. , Albitar, M. , Saliba, R.M. , Ippoliti, C. , Ma, Y.C. , Keating, M.J. & Champlin, R.E. (2004) Low‐dose alemtuzumab (Campath) in myeloablative allogeneic stem cell transplantation for CD52‐positive malignancies: decreased incidence of acute graft‐versus‐host‐disease with unique pharmacokinetics. Bone Marrow Transplantation, 33, 833–837. 14755312 10.1038/sj.bmt.1704435

[bjh13239-bib-0017] Kottaridis, P.D. , Milligan, D.W. , Chopra, R. , Chakraverty, R.K. , Chakrabarti, S. , Robinson, S. , Peggs, K. , Verfuerth, S. , Pettengell, R. , Marsh, J.C.W. , Schey, S. , Mahendra, P. , Morgan, G.J. , Hale, G. , Waldmann, H. , de Elvira, M.C.R. , Williams, C.D. , Devereux, S. , Linch, D.C. , Goldstone, A.H. & Mackinnon, S. (2000) In vivo CAMPATH‐1H prevents graft‐versus‐host disease following nonmyeloablative stem cell transplantation. Blood, 96, 2419–2425. 11001893

[bjh13239-bib-0018] Liga, M. , Triantafyllou, E. , Tiniakou, M. , Lambropoulou, P. , Karakantza, M. , Zoumbos, N.C. & Spyridonidis, A. (2013) High alloreactivity of low‐dose prophylactic donor lymphocyte infusion in patients with acute leukemia undergoing allogeneic hematopoietic cell transplantation with an alemtuzumab‐containing conditioning regimen. Biology of Blood and Marrow Transplantation, 19, 75–81. 22871557 10.1016/j.bbmt.2012.07.021

[bjh13239-bib-0019] Mackinnon, S. , Barnett, L. , Heller, G. & O'Reilly, R.J. (1994) Minimal residual disease is more common in patients who have mixed T‐cell chimerism after bone marrow transplantation for chronic myelogenous leukemia. Blood, 83, 3409–3416. 8193379

[bjh13239-bib-0020] Malladi, R.K. , Peniket, A.J. , Littlewood, T.J. , Towlson, K.E. , Pearce, R. , Yin, J. , Cavenagh, J.D. , Craddock, C. , Orchard, K.H. , Olavarria, E. , McQuaker, G. , Collin, M. & Marks, D.I. (2009) Alemtuzumab markedly reduces chronic GVHD without affecting overall survival in reduced‐intensity conditioning sibling allo‐SCT for adults with AML. Bone Marrow Transplantation, 43, 709–715. 19029965 10.1038/bmt.2008.375

[bjh13239-bib-0021] Mead, A.J. , Thomson, K.J. , Morris, E.C. , Mohamedbhai, S. , Denovan, S. , Orti, G. , Fielding, A.K. , Kottaridis, P.D. , Hough, R. , Chakraverty, R. , Linch, D.C. , Mackinnon, S. & Peggs, K.S. (2010) HLA‐mismatched unrelated donors are a viable alternate graft source for allogeneic transplantation following alemtuzumab‐based reduced‐intensity conditioning. Blood, 115, 5147–5153. 20371745 10.1182/blood-2010-01-265413

[bjh13239-bib-0022] Mohamedbhai, S.G. , Edwards, N. , Morris, E.C. , Mackinnon, S. , Thomson, K.J. & Peggs, K.S. (2012) Predominant or complete recipient T‐cell chimerism following alemtuzumab‐based allogeneic transplantation is reversed by donor lymphocytes and not associated with graft failure. British Journal of Haematology, 156, 516–522. 22171699 10.1111/j.1365-2141.2011.08944.x

[bjh13239-bib-0023] Morris, E.C. , Rebello, P. , Thomson, K.J. , Peggs, K.S. , Kyriakou, C. , Goldstone, A.H. , Mackinnon, S. & Hale, G. (2003) Pharmacokinetics of alemtuzumab used for in vivo and in vitro T‐cell depletion in allogeneic transplantations: relevance for early adoptive immunotherapy and infectious complications. Blood, 102, 404–406. 12623851 10.1182/blood-2002-09-2687

[bjh13239-bib-0024] Peggs, K.S. , Thomson, K. , Hart, D.P. , Geary, J. , Morris, E.C. , Yong, K. , Goldstone, A.H. , Linch, D.C. & Mackinnon, S. (2004) Dose‐escalated donor lymphocyte infusions following reduced intensity transplantation: toxicity, chimerism, and disease responses. Blood, 103, 1548–1556. 14576063 10.1182/blood-2003-05-1513

[bjh13239-bib-0025] Peggs, K.S. , Sureda, A. , Qian, W. , Caballero, D. , Hunter, A. , Urbano‐Ispizua, A. , Cavet, J. , Ribera, J.M. , Parker, A. , Canales, M. , Mahendra, P. , Garcia‐Conde, J. , Milligan, D. , Sanz, G. , Thomson, K. , Arranz, R. , Goldstone, A.H. , Alvarez, I. , Linch, D.C. , Sierra, J. & Mackinnon, S. (2007) Reduced‐intensity conditioning for allogeneic haematopoietic stem cell transplantation in relapsed and refractory Hodgkin lymphoma: impact of alemtuzumab and donor lymphocyte infusions on long‐term outcomes. British Journal of Haematology, 139, 70–80. 17854309 10.1111/j.1365-2141.2007.06759.x

[bjh13239-bib-0026] Perez‐Simon, J.A. , Kottaridis, P.D. , Martino, R. , Craddock, C. , Caballero, D. , Chopra, R. , Garcia‐Conde, J. , Milligan, D.W. , Schey, S. , Urbano‐Ispizua, A. , Parker, A. , Leon, A. , Yong, K. , Sureda, A. , Hunter, A. , Sierra, J. , Goldstone, A.H. , Linch, D.C. , San Miguel, J.F. & Mackinnon, S. (2002) Nonmyeloablative transplantation with or without alemtuzumab: comparison between 2 prospective studies in patients with lymphoproliferative disorders. Blood, 100, 3121–3127. 12384408 10.1182/blood-2002-03-0701

[bjh13239-bib-0027] Potter, V.T. , Krishnamurthy, P. , Barber, L.D. , Lim, Z. , Kenyon, M. , Ireland, R.M. , de Lavallade, H. , Dhouri, A. , Marsh, J.C. , Marcus, R. , Devereux, S. , Ho, A. , Pagliuca, A. & Mufti, G.J. (2014) Long‐term outcomes of alemtuzumab‐based reduced‐intensity conditioned hematopoietic stem cell transplantation for myelodysplastic syndrome and acute myelogenous leukemia secondary to myelodysplastic syndrome. Biology of Blood and Marrow Transplantation, 20, 111–117. 24216184 10.1016/j.bbmt.2013.10.021

[bjh13239-bib-0028] Rebello, P. & Hale, G. (2002) Pharmacokinetics of CAMPATH‐1H: assay development and validation. Journal of Immunological Methods, 260, 285–302. 11792397 10.1016/s0022-1759(01)00556-7

[bjh13239-bib-0029] Shah, A.J. , Kapoor, N. , Crooks, G.M. , Weinberg, K.I. , Azim, H.A. , Killen, R. , Kuo, L. , Rushing, T. , Kohn, D.B. & Parkman, R. (2007) The effects of Campath 1H upon graft‐versus‐host disease, infection, relapse, and immune reconstitution in recipients of pediatric unrelated transplants. Biology of Blood and Marrow Transplantation, 13, 584–593. 17448918 10.1016/j.bbmt.2007.01.076

[bjh13239-bib-0030] Shulman, H.M. , Sullivan, K.M. , Weiden, P.L. , McDonald, G.B. , Striker, G.E. , Sale, G.E. , Hackman, R. , Tsoi, M.S. , Storb, R. & Thomas, E.D. (1980) Chronic graft‐versus‐host syndrome in man. A long‐term clinicopathologic study of 20 Seattle patients. American Journal of Medicine, 69, 204–217. 6996481 10.1016/0002-9343(80)90380-0

[bjh13239-bib-0031] Spyridonidis, A. , Liga, M. , Triantafyllou, E. , Themeli, M. , Marangos, M. , Karakantza, M. & Zoumbos, N. (2011) Pharmacokinetics and clinical activity of very low‐dose alemtuzumab in transplantation for acute leukemia. Bone Marrow Transplantation, 46, 1363–1368. 21170091 10.1038/bmt.2010.308 PMC3191504

[bjh13239-bib-0032] Tholouli, E. , Liakopoulou, E. , Greenfield, H.M. , Shaw, B.E. , Tauro, S. , Byrne, J.L. , Dennis, M. , Burthem, J. , Lucas, G.S. , Craddock, C. , Russell, N.H. & Liu Yin, J.A. (2008) Outcomes following 50 mg versus 100 mg alemtuzumab in reduced‐intensity conditioning stem cell transplants for acute myeloid leukaemia and poor risk myelodysplasia. British Journal of Haematology, 142, 318–320. 18492100 10.1111/j.1365-2141.2008.07184.x

[bjh13239-bib-0033] Thomson, K.J. , Morris, E.C. , Milligan, D. , Parker, A.N. , Hunter, A.E. , Cook, G. , Bloor, A.J. , Clark, F. , Kazmi, M. , Linch, D.C. , Chakraverty, R. , Peggs, K.S. & Mackinnon, S. (2010) T‐cell‐depleted reduced‐intensity transplantation followed by donor leukocyte infusions to promote graft‐versus‐lymphoma activity results in excellent long‐term survival in patients with multiply relapsed follicular lymphoma. Journal of Clinical Oncology, 28, 3695–3700. 20606089 10.1200/JCO.2009.26.9100

